# ER Stress-Mediated Signaling: Action Potential and Ca^2+^ as Key Players

**DOI:** 10.3390/ijms17091558

**Published:** 2016-09-15

**Authors:** Entaz Bahar, Hyongsuk Kim, Hyonok Yoon

**Affiliations:** 1 College of Pharmacy, Research Institute of Pharmaceutical Sciences, Gyeongsang National University, Jinju 52828, Gyeongnam, Korea; entaz_bahar@yahoo.com; 2 Department of Electronics Engineering, Chonbuk National University, Jeonju 54896, Jeonbuk, Korea; hskim@jbnu.ac.kr

**Keywords:** endoplasmic reticulum stress, unfolded protein response, calcium, apoptosis, action potential

## Abstract

The proper functioning of the endoplasmic reticulum (ER) is crucial for multiple cellular activities and survival. Disturbances in the normal ER functions lead to the accumulation and aggregation of unfolded proteins, which initiates an adaptive response, the unfolded protein response (UPR), in order to regain normal ER functions. Failure to activate the adaptive response initiates the process of programmed cell death or apoptosis. Apoptosis plays an important role in cell elimination, which is essential for embryogenesis, development, and tissue homeostasis. Impaired apoptosis can lead to the development of various pathological conditions, such as neurodegenerative and autoimmune diseases, cancer, or acquired immune deficiency syndrome (AIDS). Calcium (Ca^2+^) is one of the key regulators of cell survival and it can induce ER stress-mediated apoptosis in response to various conditions. Ca^2+^ regulates cell death both at the early and late stages of apoptosis. Severe Ca^2+^ dysregulation can promote cell death through apoptosis. Action potential, an electrical signal transmitted along the neurons and muscle fibers, is important for conveying information to, from, and within the brain. Upon the initiation of the action potential, increased levels of cytosolic Ca^2+^ (depolarization) lead to the activation of the ER stress response involved in the initiation of apoptosis. In this review, we discuss the involvement of Ca^2+^ and action potential in ER stress-mediated apoptosis.

## 1. Introduction

The endoplasmic reticulum (ER) is a vital organelle in eukaryotic cells, responsible for multiple cellular activities, including synthesis, maturation, translation and folding of secretory and membrane proteins, lipid biogenesis, and the sequestration of Ca^2+^ [1,2]. The ER quality control (ERQC) system is involved in the proofreading of nascent and newly synthesized proteins in order to protect cells against the pathological accumulation of unfolded and misfolded proteins [3,4,5]. Disturbances in the cellular energy levels, the redox state, or Ca^2+^ concentrations reduce the protein folding capacity of the ER, and lead to the accumulation and aggregation of unfolded proteins, resulting in ER stress [1]. It has been reported that ER stress is triggered by disturbed ER functions, especially by the increase in protein secretion or protein misfolding [6]. ER stress leads to the activation of three ER-resident transmembrane proteins called activating transcription factor-6 (ATF6), inositol requiring protein-1 (IRE1), and protein kinase RNA-like ER kinase (PERK) [7]. All three ER stress receptors maintain direct signaling pathways that relieve ER stress by initiating the unfolded protein response (UPR). UPR is a pro-survival response, responsible for restoring normal ER functions by reducing the aggregation and accumulation of unfolded proteins [8]. Prolonged UPR activation or adaptive response failure can promote a pro-survival response to a pro-apoptotic signaling, especially in the pathological condition [7].

The term apoptosis is used interchangeably with the term programmed cell death, which represents a genetically regulated form of cell death [9]. Apoptosis plays a vital role in the elimination of cells, which is important for the processes of embryogenesis, development, and tissue homeostasis [10]. It has been reported that ER stress is a major cause affecting the initiation of apoptosis [11]. Impairment of apoptosis can lead to a variety of pathological diseases, including neurodegenerative and autoimmune diseases, cancer, or acquired immune deficiency syndrome (AIDS) [12,13,14].

Ca^2+^ is a ubiquitous and versatile intracellular second messenger involved in many signaling processes, including myofilaments contraction, secretion of hormones, growth factors, and neurotransmitters, and the modulation of metabolism, synaptic transmission, and gene transcription [15,16,17]. Loss of cellular homeostasis disrupts Ca^2+^ signaling, inducing ER stress response [15]. Ca^2+^ is a major player in the regulation of cell death [18], both at the early and late stages of apoptosis, and severe Ca^2+^ dysregulation can induce ER stress-mediated apoptosis in response to various pathological conditions [19,20,21,22].

Action potential is an electrical signal responsible for the transmission along neurons and muscle fibers conveying the information to, from, and within the brain [23]. Action potential propagation is necessary for all essential processes and functions, for example, reading a text and understanding its message, laughing and crying, thinking and feeling, hearing, and moving our muscles [24]. Currently, research is focused on the understanding of action potentials and their effects on muscle and neuronal activities. Many currently investigated diseases, including Alzheimer’s disease (AD), myasthenia gravis, and epilepsy, involve the disturbances in action potential propagation [25,26,27].

This review focuses on: (a) ER stress and UPR signal transduction pathways; (b) the role of Ca^2+^ in the ER stress and apoptosis; and (c) the involvement of action potential in ER stress-mediated apoptosis.

## 2. Endoplasmic Reticulum (ER) Stress and Unfolded Protein Response (UPR)

### 2.1. The ER

**Figure 1:**
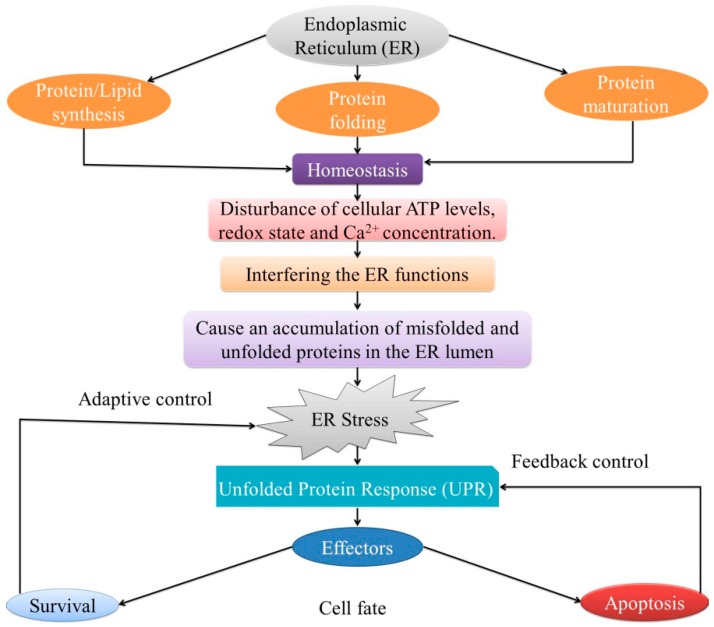
Endoplasmic reticulum (ER) Stress and unfolded protein response (UPR). ER functions include protein synthesis, maturation, and the folding of proteins, ensuring cellular homeostasis. The disturbance of cellular adenosine triphosphate (ATP) levels, redox state, or Ca^2+^ concentration affects ER functioning, causing the accumulation and aggregation of unfolded proteins, and generating ER stress, which further triggers UPR. The UPR has three major roles: in adaptive response, feedback control, and cell fate. In the adaptive response, the UPR reduces ER stress and restores ER homeostasis. The UPR signaling is inhibited through a negative feedback mechanism. Depending on the severity of the ER stress, the UPR can regulate both cellular survival and death.

ER is an essential central cellular organelle of each eukaryotic cell, which plays a vital role in the synthesis, maturation, and folding of proteins that go through the secretory pathway (Figure 1) [1]. ER ensures correct protein functioning by executing and regulating many posttranslational modifications [4,28]. Several factors, including adenosine triphosphate (ATP), Ca^2+^, and an oxidizing environment regulate the optimal protein folding through disulfide-bond formation [29]. The ERQC process assists the transit of properly folded proteins in membrane vesicles to different organelles and the surface or extracellular space of the cell [30,31]. Proper folding of newly synthesized proteins is assisted, after the translocation to the ER, by a complex network of chaperones, foldases, and cofactors [32].

### 2.2. Rough Endoplasmic Reticulum (RER) and Smooth Endoplasmic Reticulum (SER)

There are two types of the ER: rough endoplasmic reticulum (RER) and smooth endoplasmic reticulum (SER) [33]. As RER is studded with ribosomes, it is called rough and it plays a crucial role in the production of proteins, protein folding, quality control, and dispatch of proteins [33]. SER is called smooth, as it is associated with smooth slippery fats and it is not studded with ribosomes [31]. SER is involved in the production and metabolism of fats and steroid hormones [34]. ER is adjacent to the nuclear envelope and closely associated with the Golgi apparatus, and the proteins are transported directly between them, and ultimately into secretory vesicles that are transported through the cytoplasm [35].

### 2.3. ER and Protein Quality Control System

The ER plays an important role in the protein quality control by proofreading nascent and newly synthesized proteins, and mediating the degradation of unfolded or misfolded protein, which was designated as ER-associated degradation (ERAD) [3,4,5,36]. The ERAD pathway is responsible for the identification and destruction of the proteins that are unable to pass ERQC, using a proteolytic system [3,37]. Impaired ERQC functions can lead to various severe protein folding diseases, including neurodegenerative diseases, such as AD; cardiac diseases such as hypertrophy, heart failure, cardiomyopathy, and atherosclerosis; and cancer [38,39,40,41].

### 2.4. ER Stress

The ER is the primary organelle involved in signal transduction that senses homeostatic changes and provides feedback to other cellular components [42]. All proteins are usually folded into their tertiary and quaternary structures in the ER [1]. Perturbation of cellular ATP levels, Ca^2+^ concentration, or the redox state lead to the reduction in the protein-folding capacity of the ER, resulting in the accumulation and aggregation of unfolded proteins, known as ER stress (Figure 1) [43]. ER stress is induced by the excessive protein traffic and the accumulation of unfolded protein aggregates [44].

### 2.5. UPR

ER stress triggers the UPR (Figure 1), an adaptive response responsible for restoring protein homeostasis [45,46]. The UPR is mediated by three ER-localized proteins: IRE1, PERK, and ATF6. The luminal domains of these proteins bind an ER chaperone, binding-immunoglobulin protein (BiP), and are kept inactive during unstressed conditions. During ER stress, these proteins dissociate from BiP, which results in their activation [47]. The UPR has three major roles: (a) adaptive response, reducing ER stress and restoring ER homeostasis; (b) feedback control, in order to block the UPR when ER homeostasis is regained; and (c) balancing cellular survival and death through the regulation of apoptosis (Figure 1) [48,49].

### 2.6. UPR Signaling

**Figure 2:**
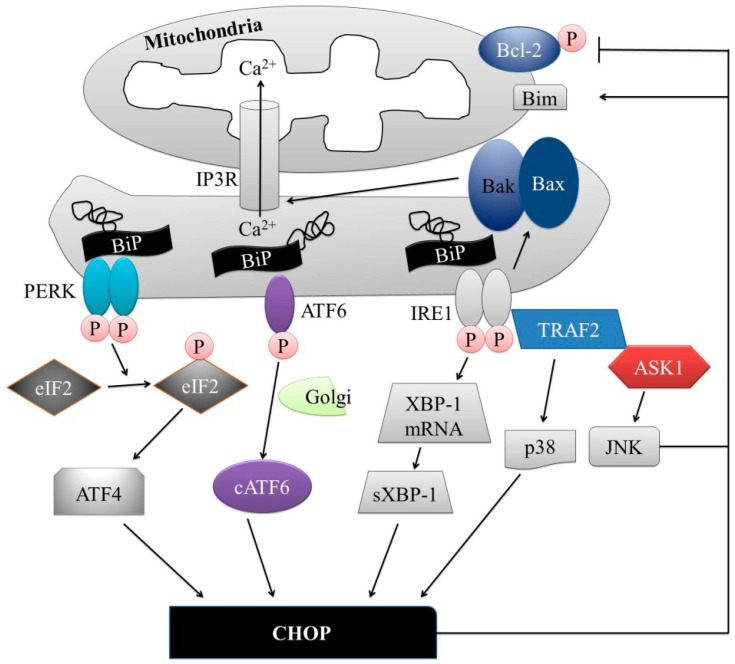
UPR mechanism. Upon the aggregation of the unfolded proteins, binding-immunoglobulin protein (BiP) dissociates from inositol requiring protein-1 (IRE1), protein kinase RNA-like ER kinase (PERK), and activating transcription factor-6 (ATF6), allowing their activation. Activated IRE1 splices X-box-binding protein 1 (XBP-1) mRNA, producing spliced XBP-1 (sXBP-1) that translocates to the nucleus and regulates the expression of C/EBP homologous protein (CHOP) transcription factor. IRE1 can recruit tumor necrosis factor receptor (TNFR)-associated factor-2 (TRAF2) and apoptosis-signaling-kinase 1 (ASK1), resulting in the downstream activation of c-Jun N-terminal protein kinase (JNK) and p38 mitogen-activation protein kinase (MAPK). Activated p38 MAPK phosphorylates and activates CHOP, whereas JNK translocates to the mitochondrial membrane, inhibiting B-cell lymphoma 2 (Bcl-2) and activating Bcl-2 interacting protein (Bim). IRE1 can activate Bcl-2 associated X protein (Bax) and Bcl-2 homologous antagonist killer protein (Bak) that induce inositol 1,4,5-triphosphate receptors (IP3Rs) to initiate the release of Ca^2+^ from the ER. Activated PERK phosphorylates eukaryotic initiation factor 2 (eIF2), which allows the translation of ATF4 through an eIF2-independent pathway, and ATF4 translocates to the nucleus and stimulates the transcription of proteins required to regain ER homeostasis. ATF6 is activated by the Golgi resident enzymes through a limited proteolysis, and it regulates the expression of CHOP. During the ER stress, all three UPR pathways result in the initiation of CHOP transcription.

As previously described, UPR is regulated by three major ER-residence proteins: IRE1, PERK, and ATF6 (Figure 2). IRE1 is a type I ER resident transmembrane protein with serine/threonine kinase activity, which can detect ER stress through its N-terminal luminal domain, and it initiates the most conserved UPR signaling pathway [50]. There are two isoforms of IRE1: IRE1α and IRE1β. IRE1α has been studied extensively as it is expressed in all cell types. The unfolded proteins accumulation in the ER induces IRE1 oligomerization in ER lumen and the autophosphorylation of the cytosolic domain of IRE1 [51]. Following the activation, IRE1 splices X-box-binding protein 1 (XBP1) mRNA, leading to a shift in the codon reading frame of this mRNA, triggering the generation of a new C-terminal domain that contains an active transactivation domain, spliced XBP1 (sXBP1) [29,30,32,52]. sXBP1 induces the upregulation of UPR-related genes involved in different functions, including protein folding, protein translocation to the ER, and ERAD [53,54]. IRE1 recruits tumor necrosis factor receptor (TNFR)-associated factor-2 (TRAF2) as well, and activates apoptosis-signaling-kinase 1 (ASK1) [55]. The activation of ASK1 leads to the activation of c-Jun N-terminal protein kinase (JNK) and p38 mitogen-activation protein kinase (MAPK) [55,56]. Activated JNK molecules translocate to the mitochondrial membrane and induce the activation of Bcl-2 interacting protein (Bim) and the inhibition of B-cell lymphoma 2 (Bcl-2), whereas p38 MAPK phosphorylation leads to the activation of transcriptional factor C/EBP homologous protein (CHOP), which causes an increased expression of Bim and death receptor 5 (DR5), simultaneously decreasing the expression of Bcl-2, which leads to the initiation of apoptosis [57,58,59,60]. Bcl-2 associated X protein (Bax) and Bcl-2 homologous antagonist killer protein (Bak) can: (a) bind to and activate IRE1; and (b) stimulate inositol 1,4,5-triphosphate receptors (IP3Rs) to induce the release of Ca^2+^ from the ER [61].

PERK is a type I ER-resident transmembrane protein responsible for the attenuation of mRNA translation, which can sense ER stress and its luminal domain partially resembles IRE1 [62,63,64]. Under normal conditions, PERK is thought to bind a chaperone protein BiP, and, following the activation, it inhibits the influx of newly synthesized proteins into the already stressed ER compartment through the inactivation of the eukaryotic initiation factor 2 (eIF2) by serine 51 phosphorylation [38]. This inhibits eIF2B, guanine nucleotide exchange factor complex that recycles eIF2 to its active GTP-bound form [39], which reduces the overload of misfolded proteins, thereby alleviating ER stress [36]. eIF2 phosphorylation also allows the translation of UPR-dependent genes, such as the ATF4, that contain various upstream open reading frames [65,66]. ATF4 induces the expression of ER stress target genes, including CHOP, growth arrest and DNA-damage-inducible 34 (GADD34), and ATF3 [67,68].

ATF6 is a type II ER resident transmembrane protein, which dissociates from BiP and translocates to the Golgi compartment under ER stress conditions for further proteolytic processing [69,70]. Two Golgi resident enzymes, site-1 protease (S1P) and site-2 protease (S2P), are involved in the proteolytic cleavage of the full-length 90-kDa ATF6 [71,72]. Afterward, the cleaved N-terminal cytosolic domain of 50-kDa cytosolic basic leucine zipper (bZIP) translocates into the nucleus and binds to the ATF/cAMP response elements (CRE) and ER stress-response elements (ERSE-1) to activate the transcription of target proteins, such as BiP, XBP-1, and CHOP [73,74,75,76].

During a prolonged ER stress, IRE1, PERK, and ATF6 can induce pro-apoptotic signaling through the activation of CHOP, which subsequently leads to the initiation of apoptosis [8,77].

### 2.7. ER Stress and Ca^2+^

Ca^2+^ is one of the most important second messengers in the cell that participates in multiple cellular activities, such as protein synthesis and secretion, contraction of muscles, gene expression, cell cycle progression, metabolism, and apoptosis [78]. Intracellular Ca^2+^ is mainly stored in the ER lumen, to ensure the proper protein-folding through the activity of Ca^2+^-binding chaperones [79]. ER controls a diversity of cellular responses and signaling transduction pathways in response to stress through the transport of Ca^2+^ in and out of ER lumen. Ca^2+^ released from the ER induces apoptosis mainly through the mitochondrial cell death [53]. Additionally, Ca^2+^ released through IP3Rs at ER and mitochondrial contact sites can promote oxidative phosphorylation, which controls ATP levels and cell survival [80]. Bax and Bak are involved in Ca^2+^-mediated ER-induced apoptosis [56], and the overexpression of Bax leads to the release of Ca^2+^ from ER and subsequent increase in the mitochondrial Ca^2+^ levels, which leads to the induction of cytochrome *c* release. Bax and Bak deficient cells release a lower amount of Ca^2+^ from ER even after the treatment with IP3 and other ER Ca^2+^-mobilizing agents [81]. Ca^2+^-binding chaperones, such as calreticulin, play an important role in the quality control and proper folding of newly synthesized proteins in the ER [73]. Therefore, ER Ca^2+^ imbalance can greatly impact the folding capacity and induce ER stress-mediated apoptosis. For example, calreticulin overexpression disrupts intracellular Ca^2+^ regulation, leading to Ca^2+^-dependent apoptosis in mature cardiomyocytes [82].

## 3. Programmed Cell Death

### 3.1. Apoptosis

Apoptosis or the process of programmed cell death is a genetically regulated form of cell death, which can be determined by morphological characteristics reflecting the underlying energy-dependent biochemical mechanisms [83]. Apoptosis is considered an important element of many cellular processes, including the changes in the normal cells, immune system development, hormone-dependent atrophy, and embryonic development [84]. Organ homeostasis is regulated by apoptosis in both physiological and pathological conditions, by modulating cell number and tissue [85,86]. The inappropriate activation of apoptosis is responsible for a variety of common pathologies [12,87]. Apoptosis maintains cell populations in tissues through a homeostatic mechanism during development and aging, and this process can be used as a defense mechanism during immune responses or in tissues damaged by disease or toxic agents [88]. In both physiological and pathological conditions, many factors can trigger apoptosis, but not all cells necessarily undergo apoptosis in response to the same factors [89]. For example, cancer chemotherapy or radiotherapy induce DNA damage in some cells, which results in the activation of apoptotic death through a p53-dependent pathway [90].

### 3.2. Apoptosis and ER Stress

ER is a vital cellular organelle that can affect cellular survival or death [43]. Recently, ER stress was identified as a major process involved in the initiation of apoptosis that leads to the development of various pathological conditions, including neurodegenerative diseases, diabetes mellitus, and infectious diseases [91,92,93]. The ER resident proteins, PERK, ATF6, and IRE1 are stimulated during the prolonged ER stress and they can activate apoptotic signaling by inducing the expression of CHOP, which acts as a major ER stress-induced apoptotic factor through the regulation of Bcl-2, Bim, and DR5 expression [58,94]. CHOP represents a common UPR transcription factor, with the binding sites for ATF6, ATF4, and XBP1 present within its regulatory genes. CHOP is considered a primarily pro-apoptotic transcription factor that induces ER stress-mediated apoptosis through the regulation of Bcl-2 family members. It was demonstrated that the upregulation of Bim is CHOP-dependent in tunicamycin-treated Michigan cancer foundation-7 (MCF7) breast cancer cells [95]. Bim expression was shown to be controlled through a combined effect of CHOP-dependent transcriptional upregulation and post-translational alteration through protein phosphatase 2α (PP2α), which increases protein stability [57]. CHOP-mediated downregulation of Bcl-2 may shift the balance of Bcl-2 family members in favor of pro-apoptotic pathway, thus ensuring propagation and execution of the apoptotic signal [69].

Prolonged activation of IRE1 promotes apoptosis as well. Recently, several studies showed that the prolonged ER stress can trigger the activation of a pro-apoptotic IRE1-TRAF2-JNK pathway, which is activated through the signal transduction between IRE1-TRAF2 and phosphorylation [6]. Phosphorylated IRE1 interacts with the adaptor protein TRAF2, leading to the activation of JNK through the initiation of a phosphorylation cascade [96].

### 3.3. Apoptosis and Ca^2+^

Ca^2+^ plays a complex regulatory role in apoptosis and it is involved in various cellular functions [16,97,98]. In almost all cell types, including neurons, an increase in intracellular Ca^2+^ concentration can induce apoptosis [99]. It regulates cell death through the pro-apoptotic transition of mitochondria [100], and Ca^2+^ overload in mitochondria induces mitochondrial swelling, through the pro-apoptotic pathway that leads to the perturbation or rupture of the outer membrane, and result in the release of mitochondrial apoptotic factors into the cytosol [18]. A crucial link between Ca^2+^ and apoptosis was established by studying the Bcl-2 family and its mechanisms of action. Bcl-2 is thought to be a central regulator of apoptosis, which can block or delay cell death in various cells, from hematopoietic cells to neural cells [101]. Overexpression of Bcl-2 can prevent the reduction of Ca^2+^ concentration in the ER, and it is also believed to reduce the amount of Ca^2+^ released from the ER [102]. It has been suggested that the kinase family plays a crucial role in the Ca^2+^-mediated apoptotic signaling, and that phospholipid-dependent serine/threonine kinases are regulated by some intracellular factors, such as diacylglycerol (DAG) and Ca^2+^ [103,104]. Ca^2+^-dependent phosphatases play a vital role in the regulation of the Ca^2+^-dependent serine–threonine phosphatase calcineurin through Bcl-2 blocking [105]. Recently, it was determined that Bcl-2 ovarian killer (Bok), Bax and Bak homolog, promotes apoptosis in response to the ER stress [106]. However, the role of Bok in ER stress-induced apoptosis remains questionable due to the insufficient experimental data [107,108].

### 3.4. Role of Apoptosis in Health and Disease

Apoptosis, compared with necrosis, is better for maintaining hemostasis. Apoptosis is one of the key mechanisms during the embryonic development of organs and tissue structures, and during cell proliferation and differentiation. Loss of the control of cell death (excess apoptosis) results in a wide range of diseases, including cancer, neurodegenerative diseases, hematologic diseases, liver diseases, and general tissue damage [109].

Cancer is usually characterized by too little apoptosis, by the dysfunction of the normal mechanisms cell cycle regulation, and with either an uncontrolled cell proliferation, and/or reduced removal of tumor cells [110]. Usually, cancer cells have a number of mutations, allowing them to avoid normal growth signals and greatly increase their proliferative potential [111]. Hypoxic conditions can induce cell death through the activity of N-terminal α-helix domain of CHOP in solid tumors, while p300 is involved in the regulation of CHOP ubiquitination [112]. In leukemia, the overexpression of Na^+^/H^+^ exchanger 1 (NHE1) can trigger ER stress-induced and CHOP-mediated upregulation of DR5 receptor expression [113]. CHOP upregulation is involved in the ER stress-induced apoptosis of B-chronic lymphocytic leukemia cells as well [114].

Neurodegenerative diseases, especially Parkinson’s disease or AD, are thought to be caused mostly by cell death and the progressive loss of neurons. Many mutations of the key functional proteins are related to the upregulation of CHOP. Increased β-amyloid production and accumulation lead to the propagation of AD, and CHOP expression can induced by increased β-amyloid levels. It was demonstrated that the treatment of neuronal cells with CHOP antisense RNA can lead to their improved survival [115].

The control of cell proliferation and apoptosis is required for the development of maternal blood vessels, which enables the establishment and maintenance of a successful pregnancy [91]. Additionally, it is believed that the apoptosis is involved in the development and progression of many autoimmune diseases. For example, the dysregulation of T-lymphocyte apoptosis can result in autoreactive T-cell entering into circulation and the onset of autoimmune disease [92].

### 3.5. ER Stress-Mediated Apoptosis: The Role of Ca^2+^

#### 3.5.1. Ca^2+^ Signaling Cascade

Ca^2+^ plays a key role in many cellular processes. Cellular homeostasis is regulated by Ca^2+^-binding enzymes and proteins, cytosolic Ca^2+^ buffers, associated with the plasma membrane and various cellular components, such as cytoplasm, nucleus, mitochondria, and cellular reticular network (i.e., ER), which maintain the physiological levels of free and bound Ca^2+^ in cells [116]. It has been well established that multiple apoptotic signaling cascades are mediated in a Ca^2+^-dependent manner [97,117].

Protein kinase C (PKC), a family of phospholipid-dependent serine/threonine kinases, is regulated by various intracellular factors, including diacylglycerol (DAG) and Ca^2+^ [103]. It was demonstrated previously that PKC blocks Ca^2+^-triggered apoptosis in human acute lymphoblastic leukemia (ALL) cells [118].

Calcineurin, Ca^2+^/calmodulin-dependent protein phosphatase, was implicated in the apoptotic signaling pathway [105]. It plays a key role in the regulation of the upstream events in Ca^2+^-activated apoptosis by inhibiting Bcl-2 [105]. Additionally, it was demonstrated that protein-folding dysfunction and chronic mitochondrial Ca^2+^ overload induced by Ca^2+^ depletion in the ER lead to induce apoptosis through Bcl-2 dependent mechanisms [119]. Recently, it was reported that Ca^2+^/calmodulin-dependent protein kinase II (CaMKII) is involved in ER stress and mitochondrial apoptosis pathway activation in the fetal alcohol syndrome (Fas) [120,121]. CaMKII/ASK1 signaling pathway is important for JNK activation and apoptosis induced by several types of stimuli [122]. Ca^2+^ mediates hyperglycemia-induced apoptosis of the in retinal capillary endothelial cells (RECs) through CaMKII-JNK-Fas pathway [123].

IP_3_R-mediated Ca^2+^ release affects many signaling pathways, including the regulation of apoptosis [124]. For example, breast and ovarian cancer susceptibility gene 1 (BRCA1) stimulates apoptosis through physical and functional interaction with IP3R [125]. Previously, it was shown that IP3Rs regulate intracellular Ca^2+^ concentration during apoptosis induced by death receptor ligation and cellular damage via the activation of cytochrome *c* [126,127,128]. Functional interaction between Bcl-2 and IP3R was implicated in the suppression of IP3R activation, which regulates IP3-induced Ca^2+^ release from the ER [129]. Recently, it was established that the overexpression of multiple inositol polyphosphate phosphatase 1 (Minpp1) can promote ER stress-induced apoptosis [130].

Ryanodine receptors (RyRs) are a family of Ca^2+^ release channels found on intracellular Ca^2+^ storage/release organelles (i.e., ER), and sarcolemmal Ca^2+^ influx or depolarization represent the signals that activate these channels [131]. Some studies have shown that the depletion of intracellular Ca^2+^ stores through the activation of RyRs can induce apoptosis [132,133], because it leads to cytosolic Ca^2+^ overload, mitochondrial dysfunction, ER stress, and the subsequent cell death through α-amino-3-hydroxy-5-methyl-4-isoxazolepropionic acid (AMPA) receptor-mediated excitotoxicity in oligodendrocytes [134].

Calnexin is involved in ER stress-induced apoptosis in the fission yeast [135]. It was previously established that calnexin knockout leads to early postnatal mouse death, and is lethal in the fission yeast as well [136]. Recently, it was reported that the regulation of calnexin subcellular localization modulates ER stress-induced apoptosis in MCF7 cells [137].

Calsequestrin is a major Ca^2+^-binding protein, which plays a role in Ca^2+^ homeostasis that extends well beyond its ability to buffer Ca^2+^ ions [137]. The overexpression of cardiac calsequestrin was reported to lead to cardiomyopathy [138].

#### 3.5.2. Ca^2+^-Activated Proteases: Caspases and Calpain

Caspase signaling cascade plays a crucial role in ER stress-induced apoptosis [139]. Apoptosis is mediated by proteases called caspases, which are activated in response to extracellular signals or upon intracellular stresses [140]. Different components of Ca^2+^ signaling pathway, cleaved by caspases, can lead to the activation of various cellular processes.

The destabilization of the N-terminal amino-acid residues (or N-degron) of protein substrates was described as the N-end rule, demonstrating that the regulation of the in vivo half-life of a protein is related to the identity of its N-terminal residue [141,142]. Recently, Arg/N-end rule pathway was shown to be a mechanistically specific repressor of programmed cell death [143]. The degradation of proapoptotic substrates (Asp-BRCA1, Leu-LIMK1, Tyr-NEDD9, Arg-Bid, Asp-Bcl-XL, Arg-BIMEL, Asp-EPHA4, Tyr-MET, Cys-TRAF1, and Cys-RIPK1) in the Arg/N-end rule pathway was demonstrated, together with the suppression of this pathway by the activated caspases [142,143]. The activation of apoptosis leads to the cleavage of Lyn tyrosine kinase by caspases, generating the N-terminal truncated LynΔN, which was shown to exert negative feedback on imatinib-induced apoptosis in chronic myelogenous leukemia (CML) K562 cells [144].

ER stress can activate caspase-12 through ER-specific apoptosis pathway in caspase-12-deficient mice [92]. In humans, caspase-4 is involved in ER stress-induced apoptosis pathway as an alternative to murine caspase-12 and both are cleaved specifically under ER stress conditions [92,145,146]. The downstream activation of caspase-12 leads to ER stress-induced apoptosis through the apoptosis protease-activating factor 1 (Apaf-1)/caspase-3 signaling pathway [147]. IP3R-1 was shown to act as a caspase-3 substrate and that IP3-induced Ca^2+^ release can be inhibited by caspase-3 dependent IP3R-1 cleavage [148,149]. Ca^2+^ release can act as a potentiation loop of apoptosis, representing a negative feedback mechanism [148,150]. Additionally, it was reported that in AD, Ca^2+^-permeable AMPA-type glutamate receptors are involved in caspase-mediated neuronal apoptosis [151]. The inactivation of AMPA receptors helps avoid Ca^2+^ overload and excitotoxic apoptosis in neurons [56,152].

Calpain is a Ca^2+^-dependent cysteine protease involved in the control of cell cycle [153], and it was shown that an increase in free cytosolic Ca^2+^ concentration triggers the activation of calpain-mediated neuronal apoptosis, leading to a spinal cord injury in rats [154]. Calpain activation due to cytosolic Ca^2+^ overload is thought to be responsible for the initiation of neuronal death [155]. Ca^2+^-activated calpain has been implicated in cell death in cultured neonatal rat cardiomyocytes and ischemic hearts as well [156]. Additionally, the activated calpain cleaves key elements of the apoptotic machinery, especially the members of the Bcl-2 family (Bcl-XL or Bid), caspase-12, and X-linked inhibitor of apoptosis (XIAP) [157,158,159]. Furthermore, nuclear calpain activates Ca^2+^-dependent signaling through the proteolysis of nuclear CaMKIV during the sustained Ca^2+^ influx in cultured neurons [160]. Exercise-induced protection against myocardial apoptosis and necrosis was shown to occur through the attenuation of calpain-mediated degradation of myocardial Ca^2+^-handling proteins [161]. Activated calpain can induce the activation of caspase-independent apoptotic pathway in adult injured motor neurons and during enterovirus 71 (EV71)-induced apoptosis of human epithelial HeLa cells [162]. Dysregulation of Ca^2+^ leads to calpain (or caspase-7)-dependent activation of caspase-12 and subsequent apoptosis [163,164]. It was reported that Ca^2+^ associated apoptosis is regulated by ER through caspase-dependent (cytochrome c/Apaf1/caspase-9) or independent (apoptosis inducing factor, AIF) mechanisms [164,165,166]. ER Ca^2+^ homeostatic alterations lead to the induction of ER stress and ER-mediated apoptosis through the activation of caspase-12 [167]. It was demonstrated that the calpain-generated C-terminal fragments of mammalian proteins represent the substrates of the Arg/N-end rule pathway involved in apoptosis [142,143]. Calpain activation as a result of the Ca^2+^ increase probably plays a major role in CYP2E1-dependent toxicity in human liver cancer cell line, HEPG2 [168]. In the retina degeneration model, Ca^2+^ influx leads to the activation of calpain, which results in caspase-3-mediated apoptosis [169].

## 4. Action Potential

### 4.1. Action Potential

**Figure 3:**
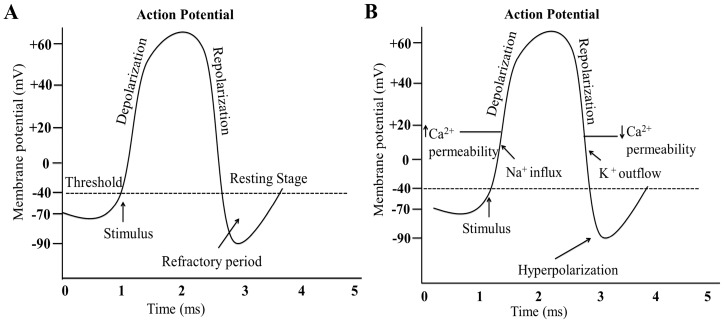
Ionic basis of action potential. (**A**) A typical action potential. The membrane potential begins at −70 mV. When a stimulus is applied after 1 ms, the membrane potential raises above −40 mV (threshold potential). If a prolonged stimulation is applied, the membrane potential rapidly rises to the peak potential (+60 mV) at time = 2 ms. Afterward, the potential rapidly drops and overshoots to −90 mV at time = 4 ms, and finally the resting potential of −70 mV is reestablished at time = 5 ms; (**B**) the role of Ca^2+^ during an action potential. Depolarization occurs due to the influx of Na^+^ ions, which causes voltage-gated Ca^2+^ channels to open. This results in the change of membrane potential first from −70 mV to −40 mV (threshold level), and then to +60 mV. When the membrane potential reaches +60 mV, Ca^2+^ channels close and voltage-gated K^+^ channels open. The efflux of K^+^ results in the repolarization of cell membrane to −70 mV and then to −90 mV (hyperpolarization).

The difference in electric potential between the exterior and the interior of a cell is called membrane potential, and, typically, its values range from −40 mV to −80 mV [170]. Action potential represents a reversal of the electric polarization (lasting for about one-thousandth of a second) of the membrane of a neuron or muscle cell (Figure 3) [171]. In neurons, signals are transmitted along the axons through the propagation of action potential, and in the muscle cell, action potential propagation leads to the muscle contractions, required for all movement [172]. All types of cells, especially neurons and muscle cells, maintain an electrochemical gradient across their membranes so that the cellular interior is negatively charged relative to the outside of cell when the membrane is at rest, which is known as the resting potential [171]. At resting condition, K^+^ concentration is higher inside and Na^+^ concentration is higher outside the cell [173]. If the cell membrane is punctured, K^+^ ions can diffuse out of the cell, while Na^+^ concentration increases inside the cell, until they reach the equilibrium of their intracellular and extracellular concentrations [173,174]. Na^+^/K^+^ pumps (or ATPases) are involved in the regulation of both Na^+^ and K^+^ concentration gradients across the plasma membrane [175]. Sodium channels are generally closed, but some Na^+^/K^+^-ATPases are open at the resting state. Therefore, K^+^ ions can exit the cell against the concentration gradient due to their positive charge, which maintains the resting membrane potential around −70–80 mV [176]. The whole system is roughly balanced because the negativity inside tends to resist further efflux of K^+^ ions. However, there is a very slight leakage of Na^+^ into the cells [175,177].

### 4.2. Ionic Basis of Action Potentials

The generation and propagation of the action potential is based on the influx of ions (Na^+^, Ca^2+^, and Cl^−^) through the ion channels, which leads to membrane depolarization (Figure 3) [178]. Inactivation of the Na^+^ channels reduces Na^+^ influx, which stops the depolarization, while K^+^ efflux is increased, which allows rapid action potential repolarization [97]. The increased K^+^ efflux is regulated by the activity of both voltage-dependent and voltage-independent K^+^ channels. The recovery of Na^+^ channels from inactivation and the slow closing of K^+^ channels following the action potential determine the membrane refractory period [179].

### 4.3. Action Potential and Ca^2+^

Ca^2+^ plays an important role in the propagation of action potential. Long-lasting Ca^2+^ channels open when the threshold (−40 mV) is reached, initiating the propagation of action potential [180,181].

Neurons contain a number of Na^+^ channels that can open and close [172]. When opened, Na^+^ influx leads to a change in the membrane potential, which further stimulates the opening of voltage-gated Ca^2+^ channels. Ca^2+^ influx leads to membrane depolarization, reaching the threshold where Ca^2+^channels close and voltage-gated K^+^ channels open, allowing the efflux of K^+^ that results in membrane repolarization [180]. When the membrane potential returns to approximately −60 mV, K^+^ channels close and Na^+^ channels open, and the action potential can be initiated again (Figure 3) [24].

## 5. ER Stress-Induced Apoptosis: Action Potential and Ca^2+^ as a Key Player

### 5.1. Physiological Role of Ca^2+^ Channel during the Initiation of Action Potential

**Figure 4:**
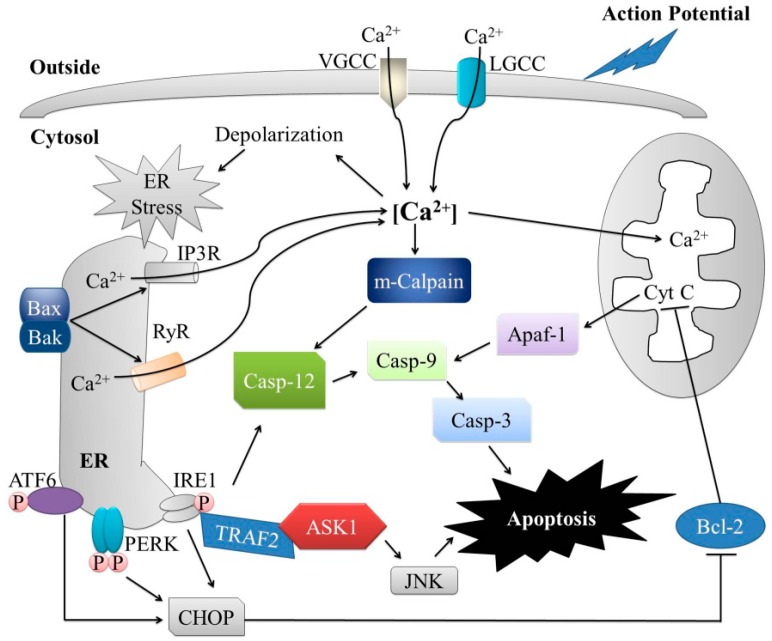
Action potential propagation induces ER stress-mediated apoptosis. During the action potential, extracellular Ca^2+^ enters into the cell through voltage-gated Ca^2+^ channels (VGCCs) and several ligand-gated calcium channels (LGCCs), while ER Ca^2+^ is released into the cytosol through IP3Rs or ryanodine receptors (RyRs). An increased level of intracellular Ca^2+^ leads to the membrane depolarization and the subsequent activation of ER stress response. Conformational changes of Bak and Bax in the ER membrane permit Ca^2+^ efflux, which activates m-calpain in the cytosol and subsequently cleaves and activates ER-resident procaspase-12, leading to the activation of the caspase cascade. Ca^2+^ is taken by mitochondria, leading to the depolarization of the inner membrane, the release of cytochrome *c*, and subsequent activation of Apaf-1/procaspase-9-regulated apoptosis. PERK and ATF6 can trigger pro-apoptotic signaling through the activation of downstream transcriptional target CHOP that inhibits the expression of Bcl-2 and thus promotes apoptosis. Activated IRE1 recruits TRAF2, which leads to the activation of ASK1/JNK and procaspase-12, subsequently activating caspase cascade.

In the resting conditions, free cytosolic Ca^2+^ levels are lower than the extracellular levels [182,183]. Upon the initiation of the action potential, Ca^2+^ levels rise through the influx of extracellular Ca^2+^ or Ca^2+^ release from intracellular stores (e.g., ER) (Figure 4) [184]. In response to the action potential initiation, Ca^2+^ channels are activated, which leads to Ca^2+^ influx into the cytosol and subthreshold depolarizing signals [185]. Both Ca^2+^ influx and Ca^2+^ release from the ER have been proposed to be apoptogenic [14].

Ca^2+^ can enter into the cell via voltage-gated Ca^2+^ channels (VGCCs) and several ligand-gated calcium channels (LGCCs), such as glutamate and acetylcholine receptors [15,186]. VGCCs are key transducers of membrane potential changes that can initiate many physiological events [185], for example, the rise in intracellular Ca^2+^ levels that is mediated by AMPA subtype of glutamate receptors, which is involved in the pathogenesis of motor neuron disease [187]. Furthermore, it was reported that the exposure of cells to glutamate receptor agonists, such as glutamate, *N*-methyl-d-aspartic acid (NMDA), and AMPA, leads to an increase in both intracellular and mitochondrial Ca^2+^ levels, causing mitochondrial depolarization and cytotoxicity in motor neurons and other spinal neurons [188,189]. IP3Rs and RyRs play critical roles in Ca^2+^-mediated signaling, including the activation of T-cell, excitation-contraction coupling, and apoptosis [190]. Recently, translocon, the ER protein import complex, or IP3Rs were suggested to contribute to the Ca^2+^ efflux from the ER [191].

### 5.2. Action Potential and Ca^2+^ in the ER Stress-Mediated Apoptosis

VGCCs are normally closed under physiological conditions or at resting membrane potential, and they are activated upon membrane depolarization [192]. Depending on cell type, the activated VGCCs allow Ca^2+^ influx into the cell, lead to the activation of Ca^2+^-dependent K^+^ channels, which results in contraction of muscles, neuronal excitation, upregulation of protein expression, or hormone or neurotransmitter release [193,194]. During the action potential, Ca^2+^ is released from the ER into the cytosol, increasing the levels of cytosolic Ca^2+^ (depolarization), which may lead to the activation of the ER stress response (Figure 4) [69,195]. The depletion of ER Ca^2+^ results in protein misfolding and chronic mitochondrial Ca^2+^ overload, which can induce apoptosis through Bcl-2-dependent mechanisms [119]. ER stress induces the localization and oligomerization of pro-apoptotic Bcl-2 proteins, Bax and Bak, at the ER, which further promotes Ca^2+^ release from the ER into the cytosol [196], through IP3Rs and RyRs [197,198,199] that are involved in the apoptotic signal transduction pathway [133,200,201,202]. The increased cytosolic Ca^2+^ concentration leads to the activation of Ca^2+^-dependent cysteine protease m-calpain, which is involved in many intracellular processes, such as signal transduction, cell cycle progression, differentiation, and apoptosis [203,204]. M-calpain was reported to cleave and activate the ER-resident procaspase-12 (Figure 4) [11,157], which contributes to the ER stress-induced cell death pathway in differentiated PC12 cells [205]. Activated caspase-12 also cleaves and activates procaspase-9 and consequently leads to the activation of caspase-3 apoptotic cascade [206].

Increased cytosolic Ca^2+^ concentrations induce Ca^2+^ uptake into the mitochondrial matrix, leading to the depolarization of the inner mitochondrial membrane and alteration of the outer membrane permeability [7]. This induces cytochrome c release and Apaf-1-dependent activation of the apoptosome, a mutisubunit protein complex that serves as a platform for caspase activation, leading to apoptosis [207]. Now, it is generally accepted that CHOP represents a major player in the regulation of the ER stress-induced apoptosis [208]. CHOP is a basic leucine zipper-containing transcription factor that suppresses the expression of Bcl-2 and activates the transcription of several genes with pro-apoptotic functions, thereby promoting the apoptosis [209,210]. The association of IRE1/TRAF2 and ER stress may help the release of procaspase-12 from TRAF2, leading to its activation [211,212]. The activated caspase-12 can directly cleave and activate caspase-9, which further induces the activation of caspase-3, resulting in apoptosis [213].

## 6. Conclusions

The ER is a dynamic organelle that plays important roles in the coordination of signaling pathways through the regulation of intracellular Ca^2+^ levels, which ensures normal cell physiological functions. Ca^2+^ molecules are stored in the ER lumen, and Ca^2+^ is involved in the regulation of various molecular chaperones, such as calcineurin, calnexin, and calreticulin, and apoptotic proteases, such as caspases and calpain, which help ERQC system determine cellular sensitivity to ER stress and apoptosis. Moreover, the ERQC system facilitates proper folding, transportation, and modification of secretory and membrane proteins, and eliminates terminally misfolded polypeptides through ERAD. ER stress-induced apoptosis was shown to present a key factor contributing to the development of several disorders, especially neurodegenerative diseases, autoimmune diseases, and cancer. An understanding of the crucial roles of Ca^2+^ and action potential in the ER stress-mediated apoptosis, and the underlying mechanisms and processes, may lead to the development of new approaches for the treatment of various diseases that occur as a consequence of ER stress and defective protein folding.
